# Unveiling Biomarkers in Head and Neck Squamous Cell Carcinoma through Bioinformatics: The Role of *SPP1* and *KRT78*

**DOI:** 10.3390/ijms252212062

**Published:** 2024-11-10

**Authors:** Jaehwan Cheon, Byoungjae Kim, Jaehyung Park, Jaemin Shin, Tae Hoon Kim

**Affiliations:** 1 Department of Otorhinolaryngology-Head & Neck Surgery, Korea University College of Medicine, Anam-ro 145, Seongbuk-gu, Seoul 02841, Republic of Korea; 2 Department of Biomedical Science, Korea University College of Medicine, Anam-ro 145, Seongbuk-gu, Seoul 02841, Republic of Korea; 3 Neuroscience Research Institute, Korea University College of Medicine, Anam-ro 145, Seongbuk-gu, Seoul 02841, Republic of Korea; 4 Mucosal Immunology Institute, Korea University College of Medicine, Anam-ro 145, Seongbuk-gu, Seoul 02841, Republic of Korea

**Keywords:** head and neck squamous cell carcinoma, bioinformatics, biomarker, *SPP1*, *KRT78*

## Abstract

Head and neck squamous cell carcinoma (HNSCC) is the most common form of head and neck cancer, ranking sixth in global cancer incidence. Identifying molecular drivers of tumorigenesis and metastasis is essential for early detection and treatment. This study analyzed gene expression profiles from three datasets (GSE6791, GSE29330, and GSE58911) to identify differentially expressed genes (DEGs) in HNSCC. Gene Ontology and Kyoto Encyclopedia of Genes and Genomes pathway analyses were employed to functionally annotate these DEGs. A protein–protein interaction (PPI) network was constructed for selecting hub genes using the STRING database. Finally, hub gene and protein expression levels were evaluated in patients with HNSCC, along with their association with overall survival. Our analysis identified twenty-eight co-DEGs comprising eight up-regulated and twenty down-regulated genes, primarily involved in extracellular matrix (ECM) organization, proteolysis, ECM disassembly, and keratinization processes. Furthermore, the PPI network revealed eight hub genes based on their high degree of connectivity. Notably, *SPP1* demonstrated up-regulation, while *KRT78* was down-regulated in HNSCC. Remarkably, the expression levels of these hub genes correlated with tumor grade, clinical cancer stage, and poor prognosis in HNSCC. Our findings hold significant clinical potential for early diagnosis and the development of novel therapeutic targets for patients with HNSCC.

## 1. Introduction

Head and neck squamous cell carcinoma (HNSCC) accounts for over 95% of malignancies in the head and neck region, making it the sixth most common cancer globally [1,2,3]. Each year, a staggering influx of approximately 890,000 new HNSCC cases arises, contributing to a toll of 450,000 deaths [4]. The advent of immune checkpoint inhibitors targeting programmed cell death protein-1 (PD-1) and programmed death-ligand 1 (PD-L1) has revolutionized treatment outcomes for several malignancies. However, in the realm of HNSCC, despite substantial advancements and considerable investment in these therapies, their efficacy remains dishearteningly low [5,6]. This may be linked to the challenges of diagnosing HNSCC, as most patients present at advanced stages, leading to poor prognoses [7]. Additionally, the five-year overall survival rate for HNSCC remains below 40% [8]. Therefore, understanding the molecular mechanisms underlying HNSCC is crucial for developing early diagnostic methods and identifying therapeutic targets.

Microarray-based bioinformatics analysis has gained increasing popularity in recent years for studying various diseases, including breast, lung, and pancreatic cancer. The aim is to unravel the complex molecular mechanisms underlying each disease and identify key genes associated with them [9,10,11], ultimately leading to the development of effective therapeutic target selection and accurate diagnostic procedures. Recently, analyzing molecular interactions has emerged as a prominent approach for identifying novel biomarkers associated with various diseases [12]. Studies accompanied by bioinformatics primarily have involved analyzing interactions among candidate molecules to identify key targets for each disease by analyzing protein–protein interaction (PPI) [9,11,13]. Despite several bioinformatics studies conducted on HNSCC [14,15,16], studies analyzing and reporting the correlation between candidate targets derived from PPI analysis and survival rates remain particularly scarce. Therefore, conducting such analyses is essential to fill this knowledge gap and improve cancer treatment outcomes.

This study aimed to uncover key genes and functional pathways involved in HNSCC by leveraging diverse clinical datasets and advanced bioinformatic analyses to deepen our understanding of HNSCC pathogenesis and identify potential biomarkers. We analyzed differentially expressed genes (DEGs) in HNSCC tumor tissues across three gene expression profiling datasets and characterized their functions through Gene Ontology (GO) and Kyoto Encyclopedia of Genes and Genomes (KEGG) pathway analyses. Furthermore, PPI networks were constructed to identify central hub genes closely associated with HNSCC. Finally, these hub genes were validated by examining protein expression levels and correlating them with overall survival rate in patients with HNSCC.

## 2. Results

### 2.1. Identification of DEGs in Three HNSCC Datasets

**Figure 1:**
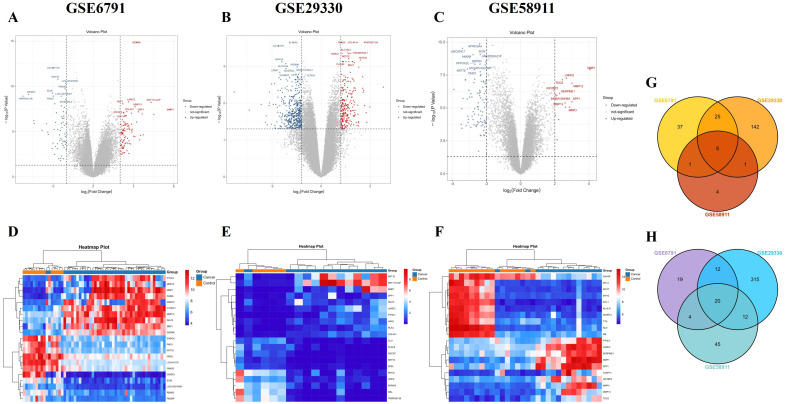
Differentially expressed gene (DEGs) and co-DEGs identification of head and squamous cell carcinoma (HNSCC) using three datasets. Volcano plots exhibiting all DEGs in each dataset (GSE6791, GSE29330, and GSE58911) are exhibited in (**A**–**C**). In the volcano maps, the red points represent up-regulated genes that were screened based on fold change greater than or equal to 2.0 with a corrected *p*-value of less than 0.05. The blue points represent down-regulated genes that were screened based on fold change less than or equal to −2.0 and a corrected *p*-value of less than 0.05. Black points indicate genes with no significant differences. Heatmap plots representing the top twenty genes in each dataset (GSE6791, GSE29330, and GSE58911) are exhibited in (**D**–**F**). In the heatmap plot, gene expression is visualized using color codes. Red indicates up-regulation, blue indicates down-regulation, and white indicates no significant change. (**G**) Eight up-regulated and (**H**) twenty down-regulated co-DEGs. Co-DEGs were identified by analyzing the cross-linking data in GSE6791, GSE29330, and GSE58911 using a Venn diagram.

Using GEO2R, we analyzed three datasets (GSE6791, GSE29330, and GSE58911) and identified a total of 63,565 up-regulated and 79,091 down-regulated DEGs. Reciprocal volcano maps for each dataset illustrate the distribution of significantly altered genes (Figure 1A–C). Representative heatmaps showcase twenty DEGs in each dataset (Figure 1D–F). Notably, cross-analysis revealed twenty-eight co-DEGs (eight up-regulated and twenty down-regulated), visualized in a Venn diagram (Figure 1G,H).

### 2.2. GO and KEGG Pathway Analysis on Up-Regulated DEGs

**Figure 2:**
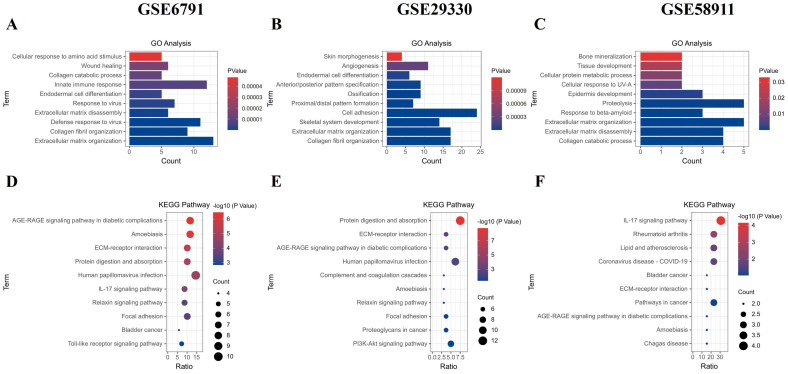
Gene Ontology (GO) terminology and Kyoto Encyclopedia of Genes and Genomes (KEGG) analyses of up-regulated DEGs.: The GO analysis categorized the up-regulated DEGs from each dataset (GSE6791, GSE29330, and GSE58911) into distinct functional groups based on their roles, as shown in (**A**–**C**). The KEGG analysis classified the up-regulated DEGs from each dataset (GSE6791, GSE29330, and GSE58911) into biochemical pathways according to their gene functions, as illustrated in (**D**–**F**).

To understand the functional implications of DEGs, we performed GO and KEGG pathway analyses for each dataset. In GSE6791, up-regulated DEGs were significantly enriched in pathways related to ECM organization, innate immune response, defense response to viruses, and other related processes (Figure 2A). GSE29330 also showed enrichment in pathways involving cell adhesion, collagen fibril organization, ECM organization, and other relevant processes (Figure 2B). Notably, GSE58911 revealed associations with ECM organization, proteolysis, collagen catabolic process, and other relevant processes (Figure 2C).

KEGG pathway analysis further highlighted specific pathways enriched with up-regulated DEGs. GSE6791 exhibited significant enrichment in diabetic complications, amoebiasis, and ECM–receptor interaction pathways (Figure 2D). Similarly, GSE29330 DEGs were enriched in protein digestion and absorption, ECM–receptor interaction, and advanced glycation end products–receptor for advanced glycation end products (AGE-RAGE) signaling pathways in diabetic complications (Figure 2E). Interestingly, GSE58911 DEGs were primarily involved in the interleukin (IL)-17 signaling pathway, rheumatoid arthritis, and lipid and atherosclerosis pathways (Figure 2F).

### 2.3. GO and KEGG Pathway Analysis on Down-Regulated DEGs

**Figure 3:**
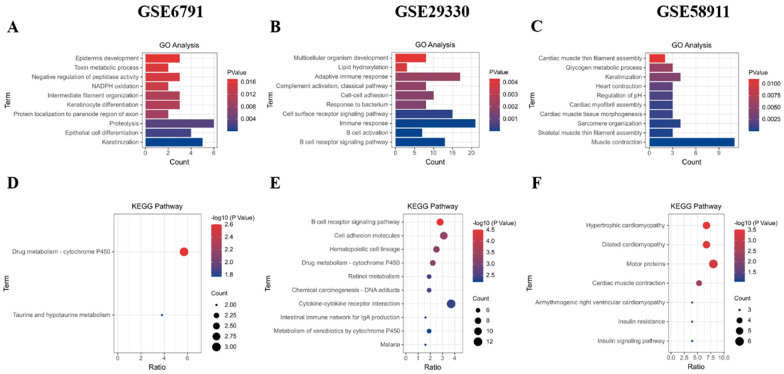
GO and KEGG analyses of down-regulated DEGs. The GO analysis categorized the down-regulated DEGs from each dataset (GSE6791, GSE29330, and GSE58911) into distinct functional groups based on their roles, as shown in (**A**–**C**). The KEGG analysis classified the down-regulated DEGs from each dataset (GSE6791, GSE29330, and GSE58911) into biochemical pathways according to their gene functions, as illustrated in (**D**–**F**).

In GSE6791, prominent enriched GO terms were associated with proteolysis, keratinization, and epithelial cell differentiation (Figure 3A). Similarly, GSE29330 down-regulated DEGs exhibited enrichment in GO terms related to immune response, adaptive immune response, and cell surface receptor signaling pathways (Figure 3B). Notably, muscle contraction, keratinization, and sarcomere organization emerged as the most frequent GO terms for down-regulated DEGs in GSE58911 (Figure 3C).

KEGG analysis further unveiled specific pathways enriched with down-regulated DEGs. GSE6791 showed significant enrichment in pathways related to drug metabolism involving cytochrome P450, taurine, and hypo-taurine metabolism (Figure 3D). Down-regulated DEGs in GSE29330 were concentrated in B cell receptor signaling, cell adhesion molecules, and hematopoietic cell lineage (Figure 3E). Interestingly, GSE58911 down-regulated DEGs were primarily associated with hypertrophic and dilated cardiomyopathy, and motor proteins (Figure 3F).

### 2.4. Identification of co-DEGs and Their GO and KEGG Pathway Analysis

**Figure 4:**
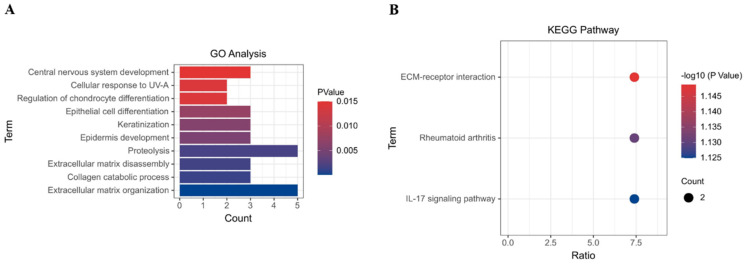
GO and KEGG analyses of co-DEGs. GO analysis (**A**) and KEGG pathway analysis (**B**) of co-DEGs. GO and KEGG pathway analyses classify co-DEGs into some functional groups and biochemical pathways based on gene character.

We next turned our attention to the overlapping co-DEGs across all three datasets and explored their shared functional roles. GO analysis revealed enrichment in terms related to ECM organization, proteolysis, and collagen catabolic processes (Figure 4A), suggesting a crucial role in ECM dynamics. Interestingly, KEGG pathway analysis highlighted significant enrichment in ECM–receptor interaction, rheumatoid arthritis, and the IL-17 signaling pathway (Figure 4B).

### 2.5. PPI Network Construction of co-DEGs and Identification of Hub Genes

**Figure 5:**
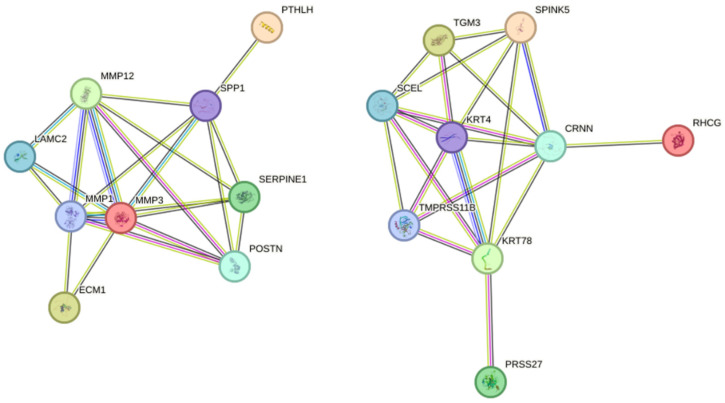
Protein–protein interaction (PPI) network of co-DEGs. Circles represent genes, and lines represent protein interactions. The results within the circles represent the protein structure. The line color represents the evidence of an interaction.

To identify hub genes potentially driving HNSCC progression, we analyzed all twenty-eight co-DEGs (eight up-regulated and twenty down-regulated) from GSE6791, GSE29330, and GSE58911 using the STRING database for PPI networks. We considered genes with connectivity exceeding six as hub genes, revealing the right promising candidates. Among these, *cornulin (CRNN)*, *matrix metalloproteinase (MMP) 1*, and *MMP3* exhibited the highest connectivity (seven), followed by *keratin (KRT) 4*, *KRT78*, *MMP12*, *sciellin (SCEL)*, and *secreted phosphoprotein (SPP) 1* (connectivity of six) (Figure 5).

### 2.6. Expression Level of Selected Hub Genes in Tumors of Patients with HNSCC

**Figure 6:**
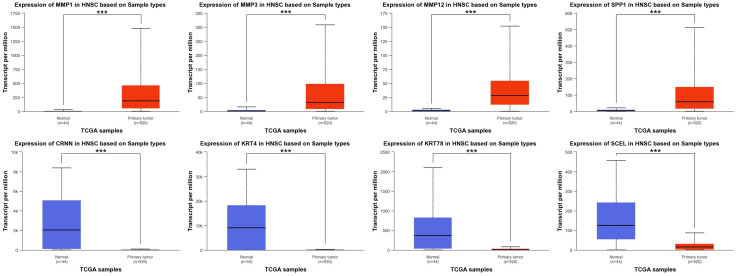
Hub genes mRNA expression in patients with HNSCC within The Cancer Genome Atlas (TCGA) database. The mRNA expression of all hub genes was significantly altered in HNSCC primary tumors compared to normal tissues. All up-regulated hub genes, including *MMP1*, *MMP3*, *MMP12*, and *SPP1*, were part of eight up-regulated co-DEGs identified in GSE6791, GSE29330, and GSE58911 (**Top**). Similarly, all down-regulated hub genes, including *CRNN*, *KRT78*, *KRT4*, and *SCEL*, were among the 20 down-regulated co-DEGs identified in the three datasets (**Bottom**). *** *p*-value < 0.001.

**Figure 7:**
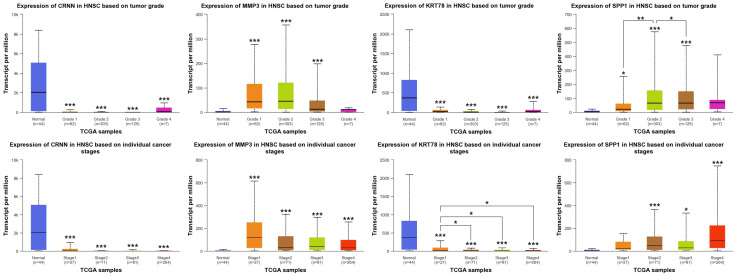
Hub genes mRNA expression based on individual cancer stage and tumor grade of patients with HNSCC. The mRNA expression of *KRT78* and *SPP1* significantly decreased and increased according to the tumor grade and individual cancer stages, respectively. * *p*-value < 0.05, ** *p*-value < 0.01, *** *p*-value < 0.001.

Using the UALCAN database, we analyzed HNSCC data from TCGA and CPTAC. As expected, *MMP1*, *MMP3*, *MMP12*, and *SPP1* displayed significantly higher expression in tumor tissues compared to normal samples (Figure 6A). Conversely, the expression levels of *CRNN*, *KRT4*, *KRT78*, and *SCEL* were down-regulated in HNSCC tumors (Figure 6B). Further analysis revealed a striking correlation between tumor grade or individual cancer stages and hub gene expression. Notably, *SPP1* expression increased markedly with higher tumor grades, while *KRT78* expression decreased progressively with advancing cancer stages (Figure 7).

**Figure 8:**
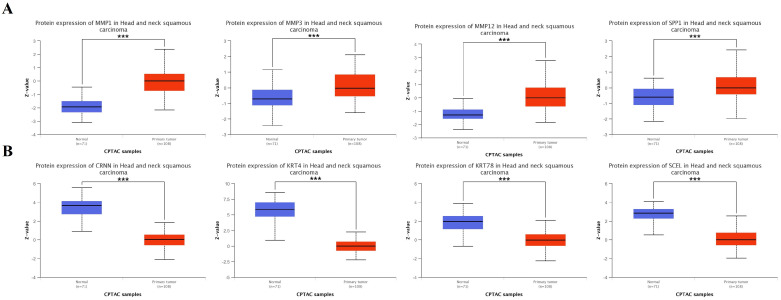
Hub genes protein expression in patients with HNSCC within Clinical Proteomic Tumor Analysis Consortium (CPTAC) database. The protein expression of all hub genes was significantly altered in HNSCC primary tumors compared with normal tissues. MMP1, MMP3, MMP12, and SPP1 were up-regulated (**A**), while CRNN, KRT78, KRT4, and SCEL were down-regulated in HNSCC (**B**), ***: *p*-value < 0.001.

**Figure 9:**
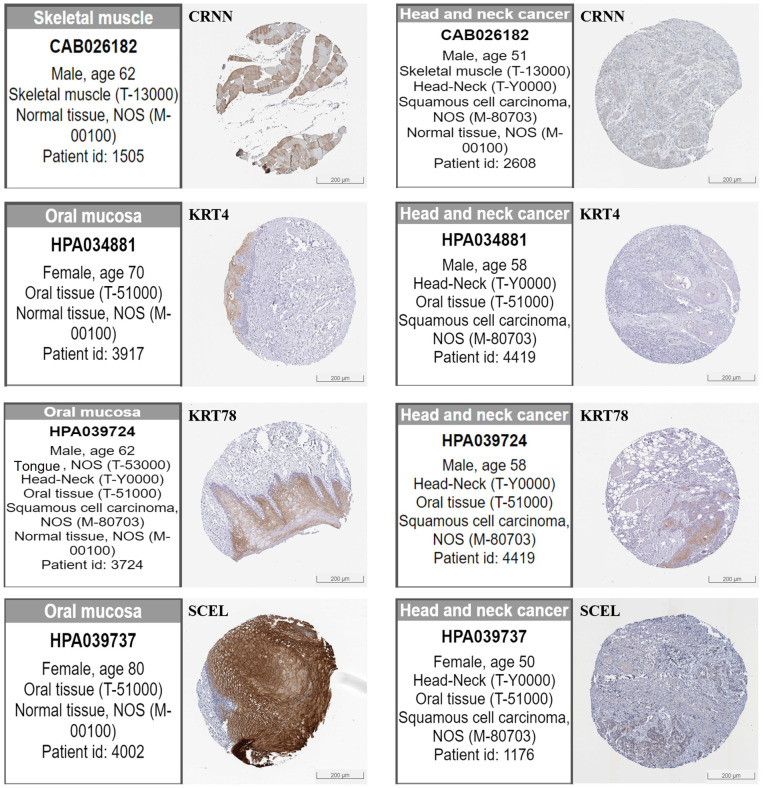
Hub genes protein expression in HNSCC tumor and normal tissue. Immuno-histochemistry results from the Human Protein Atlas (HPA) database showed lower staining intensities for CRNN, KRT78, KRT4, and SCEL in HNSCC tumor tissues than in normal tissues.

Consistent with the mRNA data, protein expression analysis using the HPA confirmed significantly elevated levels of MMP1, MMP3, MMP12, and SPP1 in HNSCC tumors compared to normal tissues (Figure 8A). Conversely, protein levels of CRNN, KRT4, KRT78, and SCEL remained down-regulated (Figure 8B and Figure 9).

### 2.7. Survival Rate Related to Hub Genes Expression in Patients with Cancer

**Figure 10:**
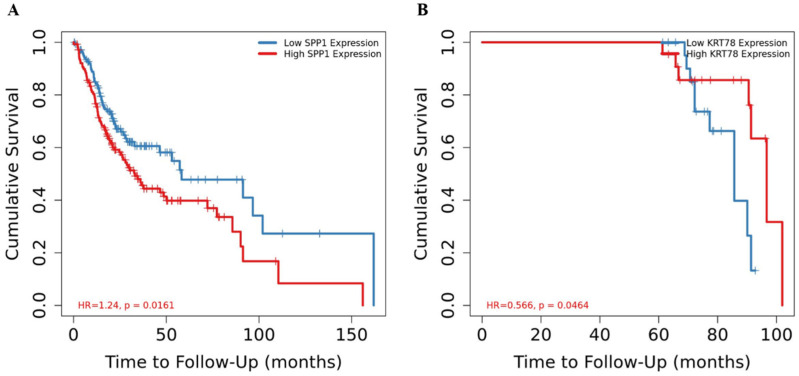
Overall survival rate analysis of the identified hub genes. Overall survival rates according to *SPP1* (**A**) and *KRT78* expression (**B**) in patients with HNSCC. High *SPP1* expression is associated with a high hazard ratio (HR) and decreased survival rate, while low *KRT78* expression is involved in decreased survival rates.

Finally, we investigated the potential link between hub gene expression and patient survival rates. Interestingly, high *SPP1* and low *KRT78* expression levels were significantly associated with poorer overall survival outcomes (Figure 10).

## 3. Discussion

HNSCC is the dominant subtype of head and neck cancer, representing over 90% of all cases [1]. Despite its high incidence and metastatic potential, early diagnosis remains challenging, and treatment options like immunotherapy lack consistent efficacy [5]. In light of these limitations, comprehensive research endeavors are crucial. In recent years, bioinformatics has offered a faster and more efficient approach to analyzing biological datasets, paving the way for novel insights [17]. In this study, we employed bioinformatics to analyze gene expression profiles from three datasets: GSE6791 (42 patients with HNSCC and 14 healthy controls), GSE29330 (13 HNSCC and 12 controls), and GSE58911 (15 HNSCC and 15 healthy controls). Our analysis identified twenty-eight co-DEGS in HNSCC, comprising eight up-regulated (Figure 1G) and twenty down-regulated DEGs (Figure 1H). Furthermore, eight hub genes were identified, four up-regulated and four down-regulated (Figure 5). Notably, among the hub genes, up-regulated *SPP1* and down-regulated *KRT78* expression significantly correlated with tumor grade, individual cancer stages, and poor survival in patients with HNSCC (Figure 7).

GO analysis revealed that co-DEGs were primarily enriched in terms related to ECM organization, proteolysis, collagen catabolic process, and ECM disassembly (Figure 4A). KEGG pathway analysis further highlighted significant enrichment in ECM–receptor interaction, rheumatoid arthritis, and the IL-17 signaling pathway (Figure 4B). This finding aligns with existing evidence associating ECM with tumor development and progression [18,19,20]. Li et al. reported that proteolytic ECM degradation, driven by elevated plasminogen activator urokinase levels, contributes significantly to HNSCC metastasis [21]. Similarly, Tanis et al. observed increased expression of ECM-related genes like MMPs, laminin, and collagen in oral squamous cell carcinoma (OSCC), another major HNSCC subtype, linking these genes to OSCC metastasis [22]. Notably, MMPs, known for their role in ECM degradation and protein up-regulation across various cancers [23], have been shown to accelerate tumor metastasis and invasion in nasopharyngeal carcinoma, another HNSCC type, through activity regulation [24]. Regarding ECM formation, overexpression of ECM-composing proteins like collagen and hyaluronic acid has been linked to increased matrix stiffness in the tumor microenvironment [25]. This aligns with our GO analysis suggesting abnormal ECM organization and catabolic reactions might contribute to cancer progression.

Our KEGG analysis further strengthens this notion by highlighting enrichment in ECM–receptor interaction consistent with Huang et al.’s findings emphasizing the crucial role of membrane receptors in recognizing ECM components in cancer [25]. Additionally, the enrichment in the rheumatoid arthritis pathway aligns with studies demonstrating enhanced immune response to Epstein–Barr virus (EBV) infection in patients with rheumatoid arthritis compared to healthy controls [26]. Given the association of EBV with both rheumatoid arthritis and HNSCC, potential similarities in immune responses between these diseases cannot be ruled out [27]. Further, clinical studies in OSCC have reported a higher frequency of T_H_17 cells and a positive correlation between IL-17 expression and tumor budding [28,29].

Through PPI network analysis, we identified four up-regulated hub genes (*MMP1*, *MMP3*, *MMP12*, and *SSP1*) and four down-regulated genes (*CRNN*, *KRT4*, *KRT78*, and *SCEL*) (Figure 5). To determine their role in HNSCC, we conducted several analyses focusing on the relationship between hub gene expression and HNSCC. Our analysis using the HNSCC dataset of TCGA database revealed significantly elevated expression of *MMP1*, *MMP3*, *MMP12*, and *SPP1* in tumor tissues compared to normal tissues (Figure 6). This finding aligns with established knowledge in several other cancers, where *MMPs* have been implicated in poor prognosis. For example, Zhang et al. observed increased MMP1 genes and protein expression in HNSCC, with overexpression positively correlating with advanced tumor size and metastasis. Similar findings have been reported for *MMP1* in colorectal cancer (CRC), where knockdown experiments confirmed its protumorigenic role [30]. Additionally, a bioinformatic study mirrored our results by demonstrating *MMP1*, *MMP3*, and *MMP12* up-regulation in CRC, highlighting the potential universal relevance of these MMPs in tumor progression [31]. This up-regulation might be related to the general tendencies of *MMPs* in cancer, where *MMP1* is significantly and almost universally up-regulated, and *MMP3* and *MMP12* show significant up-regulation in at least 10 types of cancer [32].

Conversely, our analysis revealed significantly reduced expression of *CRNN*, *KRT4*, *KRT78*, and *SCEL* in HNSCC (Figure 6). Notably, this pattern was consistent across protein expression pattern data from both the CPTAC and HPA databases (Figure 8 and Figure 9), further substantiating their potential involvement in the disease. CRNN, known for its tumor-suppressive functions in cell cycle regulation, is also down-regulated in other squamous cell carcinoma types, suggesting its broader role in epithelial malignancies [33,34,35]. Interestingly, *CRNN* down-regulation might contribute to *KRT4* down-regulation, as *CRNN* acts as a keratinocyte proliferation marker [36]. Therefore, the co-down-regulation of *CRNN* and *KRT4* might synergistically promote the progression of squamous cell carcinomas like HNSCC. Regarding *SCEL*, identified as a precursor of the cornified envelope in keratinizing tissues, our finding of its down-regulation in HNSCC aligns with observations in melanoma, where lower expression correlated with poor overall survival [37]. However, *SCEL* expression might seem to vary depending on cancer type, as it has been found in gallbladder and pancreatic cancers [38,39]. Therefore, further investigations are needed to elucidate the context-specific roles of *SCEL* in different malignancies.

Among the up-regulated hub genes, *SPP1*, also known as *Osteopontin*, stood out for its strong association with tumor grade progression, particularly grade 2 (G2). This aligns with its established role in promoting tumor development and metastasis [40]. Feng et al. demonstrated that high *SPP1* expression in patients with HNSCC correlated with lymph node metastasis and macrophage infiltration, both of which contribute to cell proliferation and invasion of tumor [41]. Interestingly, one study demonstrated that *SPP1* expression is elevated in HNSCC compared to control groups and is associated with the infiltration of CD4^+^ T-cells, macrophages, neutrophils, and dendritic cells. Furthermore, *SPP1* shows a strong association with oral leukoplakia driven by HNSCC, highlighting its potential as a diagnostic and prognostic biomarker, as well as a promising therapeutic target for HNSCC [42]. Similarly, Cho et al. showed that silencing *SPP1* in non-small cell lung cancer decreased protein levels and inhibited tumor growth [43]. Notably, elevated *SPP1* is also observed in colon, gastric, and lung cancers [40,44,45]. It is worth noting that tumor G2, characterized by intermediate growth and limited spread, might represent a tipping point for metastasis [46,47]. While both grade 1 and G2 are typically curable with treatment, targeting *SPP1*, specifically at G2, could offer an effective therapeutic or diagnostic strategy.

Among the down-regulated hub genes, *KRT78* expression displayed a significant association with individual cancer stages. These findings resonate with the established function of keratins, the intermediate filament proteins forming the cyto-skeleton. Keratins are categorized into types 1 and 2, based on their characteristics, with *KRT78* classified as type 2, alongside *KRT4* [48]. Several studies have linked abnormal keratin expression to cancer progression [49], in line with our GO analysis showing enriched keratinization in HNSCC co-DEGs. For instance, clinical studies report decreased keratin levels during the transition from normal to invasive HNSCC [50] and down-regulation of both *KRT4* and *KRT13* in OSCC [51]. Additionally, co-expression of *KRT4*, *KRT13*, and *KRT78* in the epithelium basal layer has been established [52], further supporting our findings of co-down-regulation of *KRT4* and *KRT78* in HNSCC. Interestingly, Fortier et al. showed that loss of *KRT8* and *KRT18* in epithelial cells correlated with increased *MMP2* and *MMP9* activity, promoting collective cancer cell migration [53]. This parallels our observation of decreased *KRT4* and *KRT78* alongside increased *MMP1*, *MMP3*, and *MMP12* expression in HNSCC.

## 4. Materials and Methods

### 4.1. Microarray Data

The National Center for Biotechnology Information Gene Expression Omnibus is a publicly accessible database at https://www.ncbi.nlm.nih.gov/geo, accessed on 3 August 2023. We searched for relevant keywords such as ‘head and neck cancer’, ‘head and neck squamous cell carcinoma’, and ‘HNSCC’ and identified three gene expression datasets available for download and analysis through GEO2R (https://www.ncbi.nlm.nih.gov/geo/info/geo2r.html), accessed on 3 August 2023 [54]. These datasets include GSE6791 (42 HNSCC tumor and 14 normal tissues), GSE29330 (13 HNSCC tumor and 12 normal tissues), GSE58911 (15 HNSCC tumor and 15 normal tissues). The GEO2R tool was employed to perform thorough normalization of all samples.

### 4.2. Identification of DEGs and Visualizing the Data

Using GEO2R, we analyzed DEGs between HNSCC and normal tissues across all three datasets. We identified DEGs within each dataset (GSE6791, GSE29330, and GSE58911) based on an adjusted *p*-value < 0.05 and a log_2_ fold change (log_2_ FC) threshold of |log_2_ FC| > 2. Volcano and heatmap visualizations for each DEG dataset were conducted using Hiplot (https://hiplot.com.cn/home/index.en.html, accessed on 21 August 2023) [55].

### 4.3. GO and KEGG Pathway Analysis of Up- and Down-Regulated DEGs

We utilized the Database for Annotation, Visualization, and Integrated Discovery (DAVID) web server (https://davidbioinformatics.nih.gov), accessed on 21 August 2023 for gene list annotation and functional enrichment analysis [56]. Specifically, we performed GO and KEGG pathway analyses on both up- and down-regulated DEGs using version 7.0 of the DAVID database.

### 4.4. PPI Network Build Up on Up- and Down-Regulated DEGs for Hub Genes Identification

STRING (https://string-db.org/), accessed on 21 August 2023 is a web-based database offering pre-computed protein association networks [57]. We used version 12.0 for our PPI network analysis to identify hub genes in both up- and down-regulated DEGs.

### 4.5. Analyzing the Hub Genes Expression and Their Impact on Survival Rate in Patients with Cancer

The University of Alabama at Birmingham Cancer Data Analysis Portal (UALCAN) (http://ualcan.path.uab.edu/), accessed on 11 September 2023 is a valuable resource for interactive network analysis [58]. We used UALCAN to analyze hub genes in HNSCC tumor and control tissues, along with clinical data from The Cancer Genome Atlas (TCGA; HNSCC *n* = 520 and control *n* = 44, respectively) and Clinical Proteomic Tumor Analysis Consortium (CPTAC; HNSCC *n* = 108 and control *n* = 71). Additionally, immunohistochemistry data on protein expression was obtained from the Human Protein Atlas (HPA) (https://www.proteinatlas.org/), accessed on 30 October 2023 [59]. Finally, we employed TIMER 2.0 (http://timer.cistrome.org), accessed on 30 October 2023 to analyze the expression of hub genes and their impact on survival rate within patients with HNSCC (*n* = 522) [60].

## 5. Conclusions

Our study delved into the molecular mechanisms of HNSCC progression by integrating three microarray datasets from GEO2R using R software version 4.3.1 and bioinformatic tools. We narrowed down to ten up-regulated and ten down-regulated genes most likely relevant to the disease process. Further, cross-linking analysis yielded twenty-eight candidate co-DEGs potentially implicated in HNSCC development. GO and KEGG pathways analyses revealed these co-DEGs to be primarily involved in ECM organization, disassembly, and proteolysis. These findings provide valuable insights into the biological underpinnings of HNSCC and establish a theoretical foundation for further research. Furthermore, we successfully constructed a PPI network of co-DEGs in HNSCC, identifying hub genes playing crucial roles in disease progression. By utilizing various databases, we validated the impact of hub gene up-regulation and down-regulation on HNSCC development. These results significantly enhance our understanding of HNSCC pathogenesis and the molecular mechanisms driving its occurrence and progression. Our research possesses significant clinical implications, paving the way for improved detection, treatment, and prevention of HNSCC. Notably, identified hub genes, such as *SPP1* and *KRT78*, emerge as potential targets for effective therapeutic inventions. However, thorough clinical, pharmacological, and biological studies, including in vivo and in vitro experiments, are essential to definitively confirm the functions of these hub genes and their direct involvement in HNSCC progression.

## Data Availability

The data used in this study are available from the NCBI-GEO (https://www.ncbi.nlm.nih.gov/geo, accessed on 21 August 2023), UALCAN (http://ualcan.path.uab.edu, accessed on 21 August 2023), HPA (https://www.proteinatlas.org, accessed on 21 August 2023), and TIMER 2.0 (http://timer.cistrome.org, accessed on 21 August 2023) databases, as well as reasonable requests from the corresponding author.
